# Structural basis for the facilitative diffusion mechanism by SemiSWEET transporter

**DOI:** 10.1038/ncomms7112

**Published:** 2015-01-19

**Authors:** Yongchan Lee, Tomohiro Nishizawa, Keitaro Yamashita, Ryuichiro Ishitani, Osamu Nureki

**Affiliations:** 1 Department of Biological Sciences, Graduate School of Science, University of Tokyo, 2-11-16 Yayoi, Bunkyo-ku, Tokyo 113-0032, Japan; 2 Precursory Research for Embryonic Science and Technology (PRESTO), Japan Science and Technology Agency, 4-1-8 Honcho, Kawaguchi, Saitama 332-0012, Japan; 3 RIKEN SPring-8 Center, Hyogo 679-5148, Japan

## Abstract

SWEET family proteins mediate sugar transport across biological membranes and play crucial roles in plants and animals. The SWEETs and their bacterial homologues, the SemiSWEETs, are related to the PQ-loop family, which is characterized by highly conserved proline and glutamine residues (PQ-loop motif). Although the structures of the bacterial SemiSWEETs were recently reported, the conformational transition and the significance of the conserved motif in the transport cycle have remained elusive. Here we report crystal structures of SemiSWEET from *Escherichia coli*, in the both inward-open and outward-open states. A structural comparison revealed that SemiSWEET undergoes an intramolecular conformational change in each protomer. The conserved PQ-loop motif serves as a molecular hinge that enables the ‘binder clip-like’ motion of SemiSWEET. The present work provides the framework for understanding the overall transport cycles of SWEET and PQ-loop family proteins.

## Abstract (web summary)

SWEET family proteins mediate cellular sugar efflux and exchange through a facilitative diffusion mechanism. Here, Lee *et al*. shed light on the overall sugar transport cycle of the SemiSWEET uniporter-based structures trapped in both the inward-facing and outward-facing conformations.

## Graphical abstract

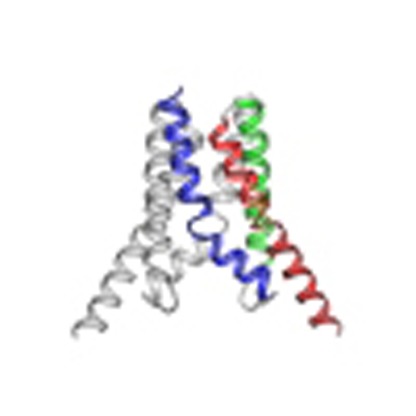

Sugar is the primary energy source used in most organisms, from bacteria to higher eukaryotes. In plants, sugar is produced in leaves during photosynthesis and is eventually delivered to various tissues throughout the body, such as roots, flowers and seeds^1^,^2^. Therefore, sugar translocation is a crucial process in plants and various membrane transporters facilitate this process. In human, the GLUT and SGLT families are the major players in sugar transport^3^. Their transport mechanisms have been well elucidated by the crystal structures of the human and bacterial transporters of these family members^4^,^5^,^6^,^7^,^8^,^9^,^10^,^11^,^12^.

Recently, the SWEET family was identified as a new class of sugar transporters that are responsible for important physiological events in plants and animals^13^. Notably, about 20 SWEET genes have been identified in plants, in which they participate in fundamental processes, including phloem transport, nectar production and pollen development^14^,^15^,^16^,^17^,^18^,^19^,^20^, as well as in plant–microbe interactions^21^,^22^,^23^. The SWEETs mediate the facilitative diffusion of sugars, by allowing solute permeation across biological membranes down a concentration gradient^13^. SWEETs possess seven transmembrane (TM) helices, consisting of a pair of 3TM repeats and an additional helix that spans the membrane to connect these two repeats, resulting in the ‘3+1+3’ arrangement of TM helices^13^. Their bacterial homologues, the SemiSWEETs, have only three TM helices. Previous studies have shown that the SemiSWEETs assemble into dimers to function as sugar transporters, and thus they are considered to be an ancestral form of the SWEETs^24^,^25^.

Phylogenetic studies revealed that the SWEET and SemiSWEET families are related to the PQ-loop family, including lysosomal amino acid exporters, whose dysfuction was reported to cause lysosomal storage diseases, such as cystinosis^26^,^27^,^28^. The members of the PQ-loop family share the same ‘3TM repeats’ architecture and possess a signature motif consisting of Pro-Gln residues (PQ-loop motif) on the first TM helix of each 3TM repeat. The first proline residue is conserved in both the SemiSWEETs and SWEETs, while the second glutamine residue is conserved in only the SemiSWEETs (Supplementary Fig. 1), suggesting the critical role of the proline residue in their transport mechanisms. Recently reported structures of the bacterial SemiSWEETs in the outward-open and occluded conformations revealed that they form dimers, to create a sugar translocating pathway at the dimer interface^25^. However, the lack of the inward-open conformation has hampered the understanding of the conformational changes occurring during the transport cycle. Furthermore, the mechanistic significance of the conserved PQ-loop motif has remained still elusive. Here we report the crystal structures of SemiSWEET from *E. coli* in the inward-open and outward-open states. The structures reveal the molecular mechanism of the overall transport cycle.

## Results

### Sucrose transport activity of *E. coli* SemiSWEET

**Figure 1:**
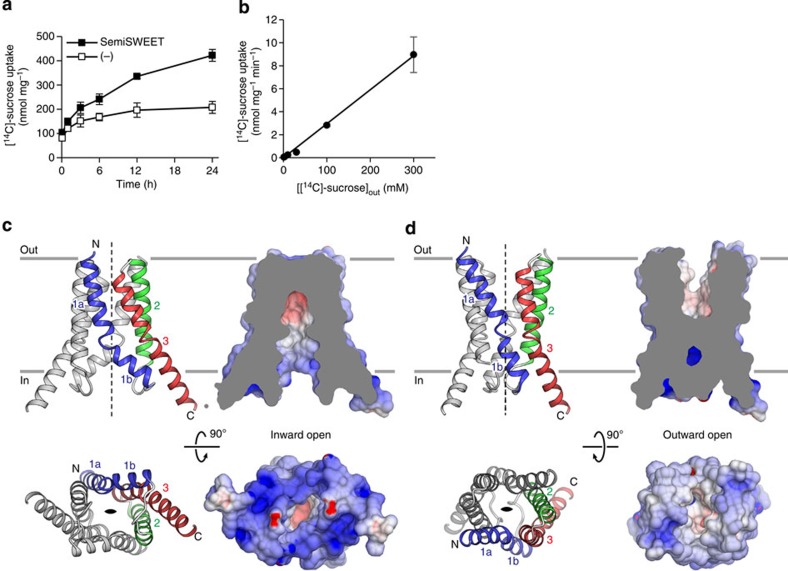
Structure and function of SemiSWEET. (**a**) Time course of [^14^C]-sucrose uptake by proteoliposomes containing EcSemiSWEET (solid black squares) or empty control liposomes (open black squares) (mean± s.e.m., *n*=6). (**b**) Plots of the sucrose uptake rate versus the extra-liposomal sucrose concentration (mean±s.e.m., *n*=3). (**c**) Overall structure of the inward-open SemiSWEET dimer, viewed parallel to the membrane (upper) or from the intracellular side (lower). In the ribbon representations (left), TM1, TM2, and TM3 of one protomer are coloured blue, green and red, respectively, and the other protomer is coloured grey. In the surface representations (right), the dimer is coloured according to the surface electrostatic potential. The cut-away surface is shown in the parallel view. The twofold axis is indicated by dashed lines and an almond-shaped symbol. (**d**) Overall structure of the outward-open SemiSWEET dimer, viewed parallel to the membrane (upper) and from the extracellular side (lower), coloured as in **c**.

We screened the bacterial SemiSWEETs and identified *E. coli* SemiSWEET (EcSemiSWEET) as a suitable candidate for structural studies. EcSemiSWEET possesses the conserved Pro-Gln motif and shares 36% sequence identity and 57% similarity with *Bradyrhizobium japonicum* SemiSWEET, which was previously characterized as a sucrose uniporter (Supplementary Fig. 1)^24^. To test the sucrose transport activity of EcSemiSWEET, we reconstituted the purified protein into liposomes and measured [^14^C]-sucrose uptake into the proteoliposomes. The proteoliposomes containing EcSemiSWEET showed slow but significant [^14^C]-sucrose uptake, as compared with the control empty liposomes (Fig. 1a). The unusually slow uptake of EcSemiSWEET compared with those of eukaryotic SWEET transporters suggests that sucrose might not be the physiological substrate. Nonetheless, our transport assay confirmed the sucrose transport activity of EcSemiSWEET. The rate of [^14^C]-sucrose uptake increased linearly according to the extraliposomal sucrose concentration and was not saturated even up to 300 mM, indicating the low-affinity binding of EcSemiSWEET to sucrose (Fig. 1b). Previous studies also reported the low affinities of the plant SWEETs for sugars^13^,^14^, suggesting a common transport mechanism.

### Overall structure

We crystallized EcSemiSWEET by the lipidic cubic phase (LCP) method and obtained crystals belonging to the space group *P*2_1_2_1_2 (Crystal-I). The structure was determined by the Se-Met single-wavelength anomalous dispersion (Se-Met SAD) method and subsequently refined to 2.0 Å resolution against the data set from a native crystal (Table 1). The crystallographic asymmetric unit contains three EcSemiSWEET molecules. Two molecules assemble to form a dimer and the other molecule forms a similar dimer with its crystallographic symmetry molecule related by a twofold axis (Supplementary Fig. 2). As the two dimers are structurally almost identical, with a root mean squared deviation value of 0.55 Å over all Cα atoms, we describe the non-crystallographic dimer of EcSemiSWEET. The two protomers are arranged with identical membrane topologies, with the central twofold axis perpendicular to the membrane (Fig. 1c). Each protomer comprises three TM helices (TM1, TM2 and TM3). TM1 is largely kinked at Pro21 in the conserved PQ-loop motif and separated into two segments (TM1a and TM1b). The short TM2 helix is almost entirely buried inside the membrane, while the long TM3 helix protrudes into the aqueous environment on the intracellular side. A total of six TM helices from two protomers create a central cavity that is widely open to the intracellular side (Fig. 1c). This cavity penetrates into the dimer core, but is completely occluded from the extracellular side and the lipid environment by the tight association of the extracellular regions and the surrounding TM helices, respectively. Hence, we designated this structure as the inward-open conformation.

We also obtained a crystal belonging to the different space group *C*2 (Crystal-II), and the structure was determined at 3.0 Å resolution by molecular replacement, using a protomer in Crystal-I as the search model. The asymmetric unit contains four EcSemiSWEET molecules organized into two separate dimers (Supplementary Fig. 2). Whereas the conformation of one dimer is almost identical to that of the inward-open state in Crystal-I, the other dimer adopts a markedly different conformation (Fig. 1d and Supplementary Fig. 2). In this alternative conformation, the extracellular halves of the protomers are separated from each other, while the intracellular halves approach the central axis (Fig. 1d). Consequently, the central cavity formed by the dimer is open towards the extracellular side. This structure is similar to the recently reported structure of the outward-open conformation of the SemiSWEET from *Vibrio sp.*^25^, with a root mean squared deviation value of 2.11 Å for Cα atoms of 164 aligned residues (Supplementary Fig. 3). Hence, we designated this structure as the outward-open state. We now describe the first crystal structure of the inward-open conformation of a SemiSWEET family member, as well as the structure of the same transporter in the outward-open conformation. The structures have enabled us to discuss the detailed conformational changes associated with the sugar transport cycle.

### Putative substrate-binding pocket

**Figure 2:**
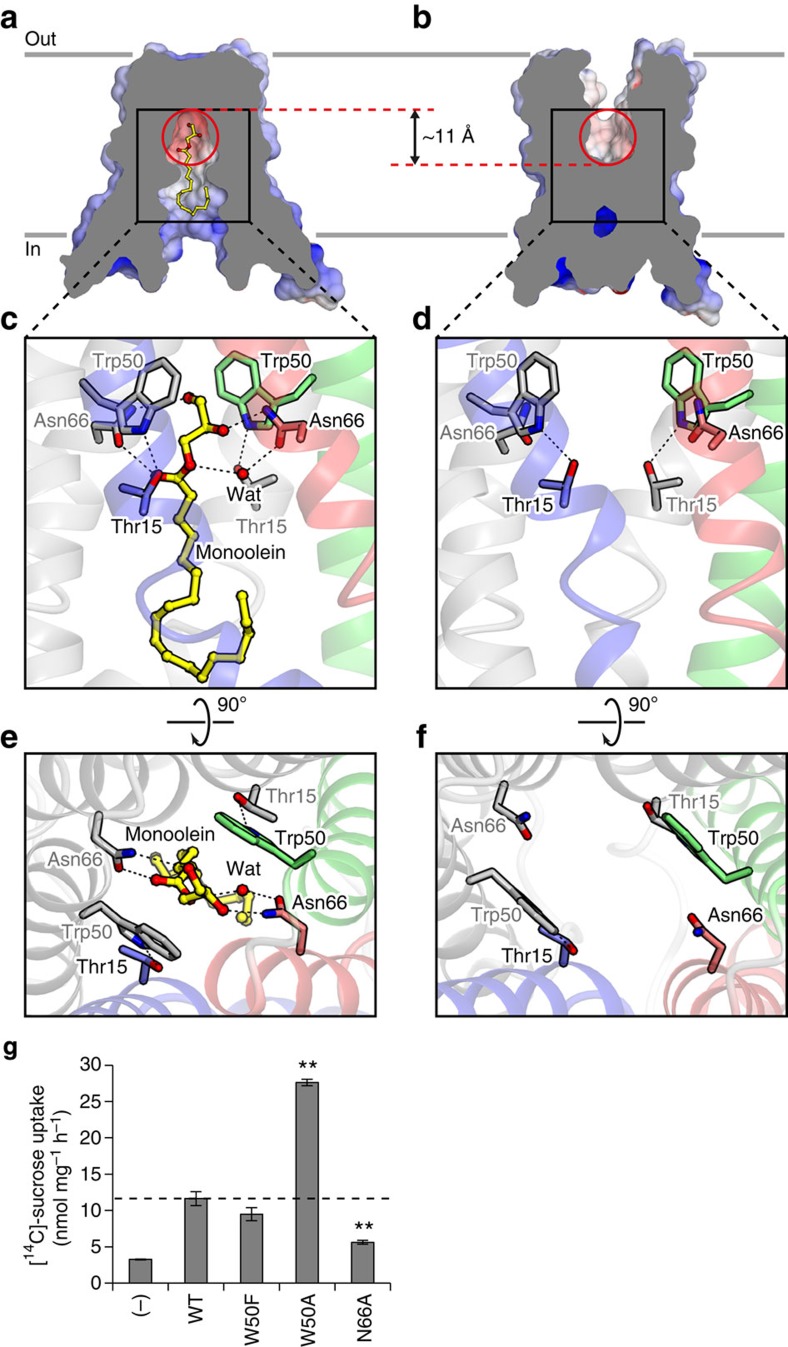
Putative substrate-binding pocket. (**a**,**b**) Cut-away surface representations of the inward-open (**a**) and outward-open (**b**) conformations. The position of the putative substrate-binding pocket is indicated by red circles. The monoolein molecule is shown as a ball-and-stick model. (**c**–**f**) Close-up views of the putative substrate-binding pocket in the inward-open (**c**,**e**) and outward-open (**d**,**f**) conformations. The residues constituting the pocket are shown as stick models. Hydrogen bonds are depicted as black dotted lines. (**g**) Sucrose uptake by the SemiSWEET mutants in the liposome assay (mean±s.e.m., *n*=3). Significant differences from the wild-type value (WT) are indicated by asterisks (**P<0.01, Student’s *t*-test).

The largely open cavities on the intracellular and extracellular sides in the inward-open and outward-open conformations, respectively, suggested that the substrate sugar is translocated along the central axis of the EcSemiSWEET dimer, accompanied by a conformational change (Fig. 2a,b). The high-resolution structure of Crystal-I revealed the presence of a monoolein molecule occupying the cavity of the inward-open state (Supplementary Fig. 4). The glycerol head group of the monoolein molecule is located in the pocket formed by symmetrically arranged residues in the dimer core. Within the pocket, the hydroxyl group of the glycerol head group forms direct and water-mediated hydrogen bonds with the side chains of Asn66 (Fig. 2c,e). In addition, the glycerol head group is sandwiched by the aromatic rings of the Trp50 side chains, which are stabilized by the side chains of Thr15. Given that glycerol and sugar share similar polyol moieties, the glycerol head group of the bound monoolein molecule is likely to be mimicking the sugar substrate. This pocket has a width of ~8–9 Å and a length of 11 Å along the central axis, and is suitable for accommodating a saccharide or disaccharide. Furthermore, in the outward-open state, this pocket is exposed to the extracellular environment, while the arrangement of the lining residues is preserved (Fig. 2d,f). Together, these observations implicated this central pocket as the binding site for transport sugars.

To investigate the functional role of this putative substrate-binding pocket, we introduced mutations to Trp50 and Asn66, and measured the sucrose uptake activities of these mutants (Fig. 2g). The N66A mutation significantly decreased the sucrose uptake activity, suggesting that the hydrophilic moiety of the Asn66 side chain is important for sucrose binding. In contrast, the W50F mutation did not affect the sucrose uptake and the W50A mutation actually greatly increased the sucrose uptake activity, suggesting that Trp50 is not essential for sucrose binding. This result is not consistent with the recent report showing the crucial roles of the equivalent tryptophan residues of *Leptospira biflexa* SemiSWEET and *Arabidopsis thaliana* SWEET1 in glucose transport^25^. This inconsistency might be due to the different size of a substrate used for the transport assay. As the disaccharide sucrose has a larger molecular size than the monosaccharide glucose, the increased sucrose uptake activity of the EcSemiSWEET W50A mutant might be attributed to the enlargement of the pocket, by the replacement of the bulky Trp residue with a smaller Ala residue, which probably promotes sucrose entry into the pocket. Thus, both our results and the previous studies suggest that the transported sugars are accommodated in the central pocket lined by Trp50 and Asn66.

As compared with other sugar transporters, such as human GLUT1, in which five residues are involved in hydrogen-bonding interactions with glucose^9^, EcSemiSWEET probably has fewer hydrophilic residues for substrate binding, consistent with its low-affinity transport in the liposome assay (Fig. 1b). Supporting this notion, even in the presence of 200 mM sucrose, the electron density for the monoolein molecule still resided in the putative substrate-binding pocket (data not shown). The Trp50 and Asn66 residues are highly conserved among the SemiSWEET and SWEET families (Supplementary Fig. 1), suggesting that the two families share substrate-binding pockets with similar architectures. Thus, our structural and functional analyses may provide insight into the low-affinity transport by the SWEET transporters.

### Molecular hinge of PQ-loop motif

**Figure 3:**
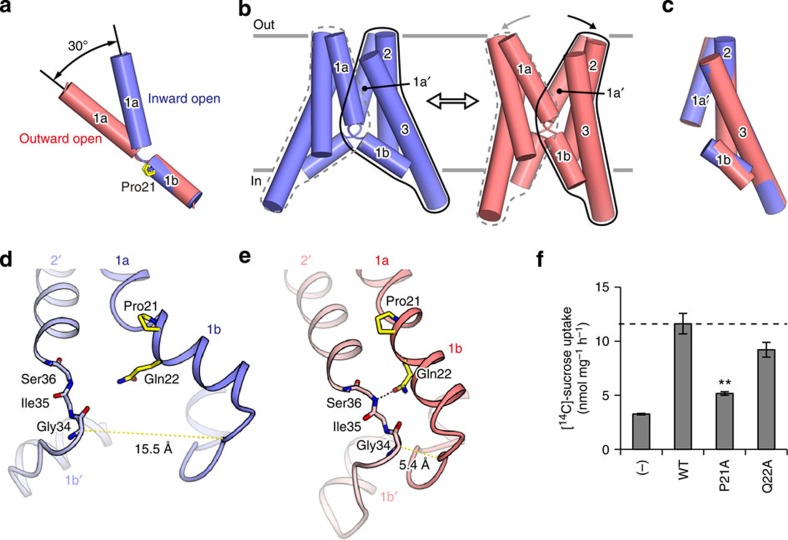
Conformational change of EcSemiSWEET. (**a**) Superimposition of TM1 between the two conformations. The inward-open and outward-open conformations are coloured blue and pink, respectively. The Pro21 residue is shown as a stick model. (**b**) Structural comparison of the overall structures between the two conformations, coloured as in **a**. The structural unit consisting of TM1b, TM2, TM3 and TM1a’ (from the adjacent protomer) is enclosed by black lines, with its counterpart enclosed by grey dotted lines. The relative rotation of the structural units is illustrated by the arrows on the top of the outward-open dimer. (**c**) Superimposition of the structural units between the two states, coloured as in **a**. (**d**,**e**) Close-up views of the PQ-loop motif in the inward-open (**d**) and outward-open (**e**) conformations, coloured as in **a**. The hydrogen bond is depicted as a black dotted line. The distance between the Gln22 side chain and the Gly34 main chain in the outward-open conformation is 4.9 Å. (**f**) Sucrose uptake by SemiSWEET mutants in the liposome assay (mean±s.e.m., *n*=3). A significant difference from the wild-type value (WT) is indicated by asterisks (**P<0.01, Student’s *t*-test).

A structural comparison of the individual protomers in the inward-open and outward-open conformations revealed an ~30° kink between TM1a and TM1b, at Pro21 in the PQ-loop motif (Fig. 3a). Furthermore, a structural comparison of the dimers in the two distinct conformations revealed the relative rotational motion of the two symmetrical helix bundles, consisting of TM1b, TM2 and TM3 from one protomer and TM1a’ from the other protomer (Fig. 3b). Each helix bundle from the two conformations can be superimposed well, suggesting their rigid body movement (Fig. 3c). This conformational change resembles a ‘binder clip’, in which the opening of the extracellular cavity is coupled with the closing of the intracellular cavity, with the kink at the PQ-loop motif serving as a molecular hinge (Fig. 3b). In addition, Gln22 in the PQ-loop motif interacts with the main chain atoms of the residues on the intracellular loop connecting TM1 and TM2, at the position immediately next to TM2 (Fig. 3d,e). In the outward-open state, the oxygen atom of the Gln22 side-chain hydrogen bonds with the backbone amide group of Ser36 (Fig. 3e and Supplementary Fig. 5). In the inward-open conformation, although the side chain of Gln22 is not within hydrogen-bonding distance with the equivalent residue, it still points towards the backbone carbonyl of Gly34 on the same loop and retains within a close distance (Fig. 3d). In contrast, the cytoplasmic end of TM1b moves largely apart from the adjacent protomer. The Cα distance between Asn31 of one protomer and Gly34 of the adjacent protomer is about 10 Å longer in the inward-open conformation than in the outward-open conformation. These observations suggest that Gln22 may stabilize the hinge by bridging the two helix bundles to allow for a dynamic structural change in TM1b and the following cytoplasmic loop. Similar interactions are also formed in the previously reported outward-open and occluded SemiSWEET conformations^25^, suggesting their conserved roles in the transport mechanism.

To verify the functional importance of the PQ-loop motif, we introduced alanine mutations to Pro21 and Gln22, and then measured the sucrose uptake by these mutants (Fig. 3f). The P21A mutation, which was expected to decrease the conformational flexibility of TM1, significantly decreased the sucrose uptake activity almost to the level of the control empty liposomes, thus demonstrating the essential role of this conserved proline residue in sucrose transport. In contrast, the Q22A mutant showed slightly decreased sucrose uptake activity as compared with that of the wild type, indicating the less important role of Gln22 in sucrose transport, which is consistent with the fact that this glutamine residue is not conserved in the SWEETs (Supplementary Fig. 1). Together, our structural and functional analyses indicated that the proline and glutamine residues in the PQ-loop motif serve as a flexible hinge, thereby enabling the binder clip-like motion of SemiSWEET. As this proline residue is highly conserved in the SWEETs and the PQ-loop family transporters^26^,^27^ and its significance for the transport function *in vivo* has been demonstrated^24^,^29^, the binder clip-like motion of EcSemiSWEET is likely to be conserved in all of these transporters.

A previous report suggested that the conformational change of SemiSWEET is mediated by the rigid-body movement of each protomer, by analogy to other well-studied transporters, such as the MFS and ABC transporters^25^. In contrast, our crystal structures in the outward-open and newly determined inward-open conformations unveiled the unexpected intramolecular conformational change in each protomer, which consequently evokes the binder clip-like motion with the PQ-loop hinge.

### Extracellular and intracellular gates

**Figure 4:**
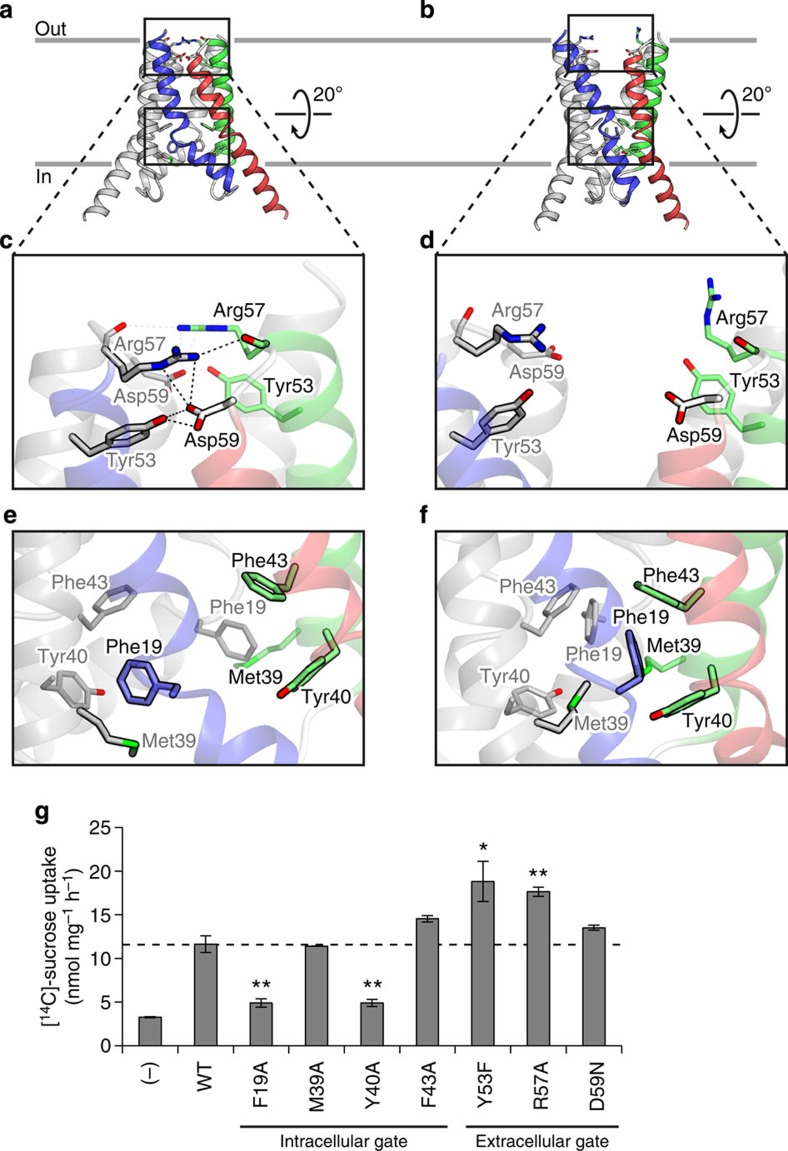
Extracellular and intracellular gates. (**a**,**b**) Ribbon representations of the inward-open (**a**) and outward-open (**b**) states, showing the positions of the intracellular and extracellular gates. The residues constituting the gates are shown as stick models. (**c**,**d**) Close-up views of the extracellular gate in the inward-open (**c**) and outward-open (**d**) states. The hydrogen-bonding and salt bridge interactions are depicted as black dotted lines. (**e**,**f**) Close-up views of the intracellular gate in the inward-open (**e**) and outward-open (**f**) states. (**g**) Sucrose uptake by SemiSWEET mutants in the liposome assay (mean±s.e.m., *n*=3). Significant differences from the wild-type value (WT) are indicated by asterisks (*P<0.05, **P<0.01, Student’s *t*-test).

The structures of the inward-open and outward-open conformations revealed the presence of two distinct gates on the extracellular and intracellular sides, which restrict the accessibility of the substrate-binding pocket (Fig. 4a,b). The extracellular gate is formed by the amino acid residues on a loop connecting TM2 and TM3 (Fig. 4c,d). In the inward-open conformation, Tyr53, Arg57 and Asp59 interact with the equivalent residues of the adjacent protomer; Asp59 forms a salt bridge with Arg57 and hydrogen bonds with Tyr53, and the Arg57 side-chain hydrogen bonds with the Arg57 main-chain carbonyl group (Fig. 4c). These interactions completely seal off the substrate-binding pocket from the extracellular environment. In contrast, in the outward-open conformation, these interactions are not observed, owing to the outward movements of TM2 and TM3 (Fig. 4d).

On the opposite side of the membrane, the intracellular gate is formed by hydrophobic residues on TM1 and TM2 (Fig. 4e,f). In the outward-open conformation, Phe19, Met39, Tyr40 and Phe43 of the two protomers form a cluster of aromatic and hydrophobic residues through van der Waals interactions (Fig. 4f). These interactions seal off the substrate-binding pocket from the intracellular environment (Fig. 4b,f). In contrast, in the inward-open state, these hydrophobic residues are separated from each other, thereby creating a cytoplasmic cavity that allows substrate access to the substrate-binding pocket (Fig. 4e). Overall, these observations suggested that the extracellular and intracellular gates restrict the central translocation pathway and determine the accessibility of the substrate-binding pocket.

The binder clip-like motion of EcSemiSWEET implied that the opening and closing of the intracellular and extracellular gates are closely related. To investigate the functional significance of these gates, we created various mutants of the residues constituting these gates and measured their sucrose transport activities (Fig. 4g). Y53F and R57A, which would disrupt the hydrogen-bonding or salt bridge interactions in the extracellular gate, showed significantly increased activities. In contrast, F19A and Y40A, which would weaken the hydrophobic interactions in the intracellular gate, showed significantly decreased activities. These results revealed that the defects in the extracellular and intracellular gates have the opposite effects on the sucrose transport (Fig. 4g). These opposite effects could be explained on the basis of the different preferences of the two conformations of EcSemiSWEET, as follows. The temperature factors for the two conformations in Crystal-II implied that the outward-open conformation is inherently less stable than the inward-open conformation, suggesting that EcSemiSWEET probably prefers the inward-open conformation in the lipid environment (Supplementary Fig. 6). Therefore, the mutation in the extracellular gate may result in the release of the extracellular ‘lock’, which preferentially captures EcSemiSWEET in the inward-open state. The observed increased sucrose uptake activities of the mutants are likely due to the facilitated state transition between the inward-open and outward-open conformations, by disrupting the interactions in the extracellular gate (Fig. 4g). In contrast, the defects in the intracellular gate may arrest EcSemiSWEET in the rather stable inward-open state, thus resulting in the lower sucrose uptake activities (Fig. 4g).

**Figure 5:**
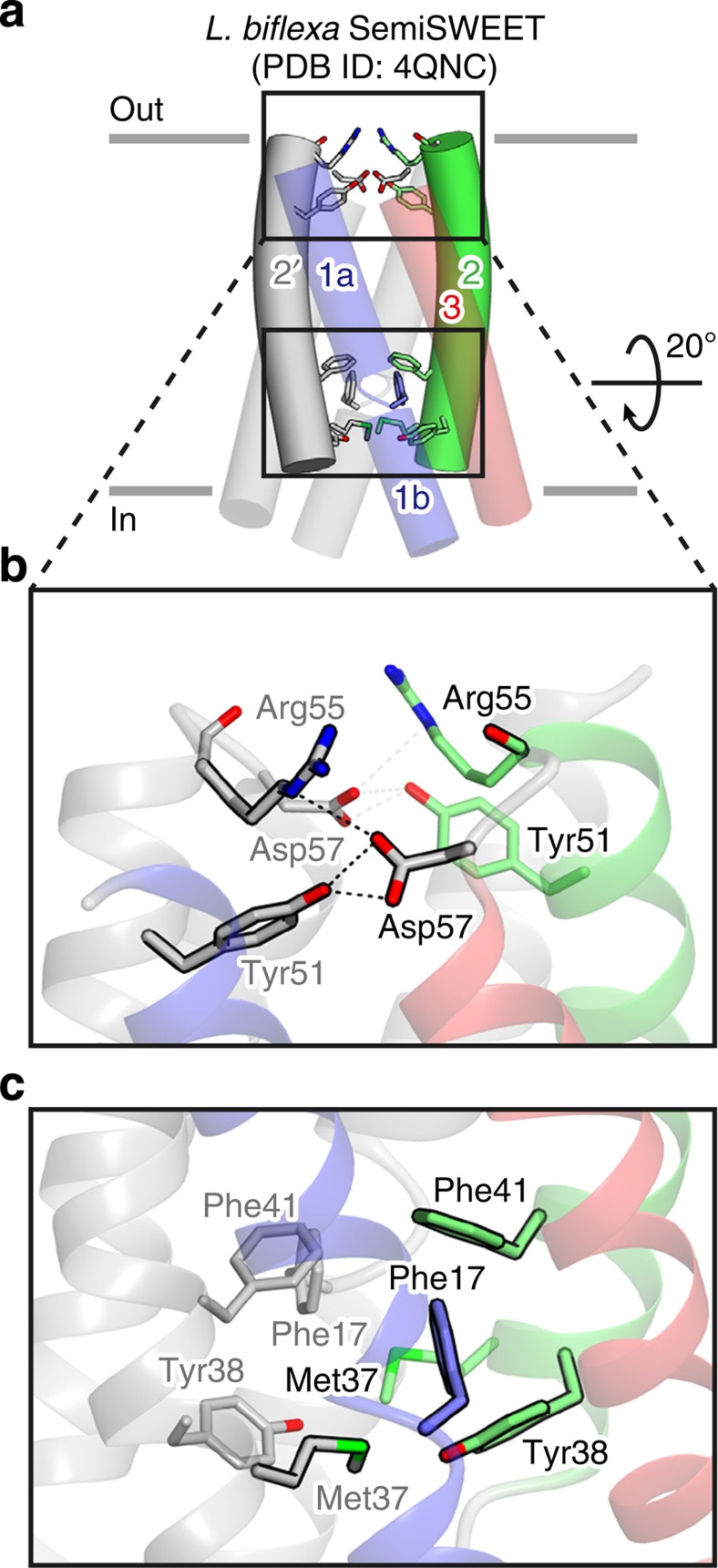
Occluded conformation of LbSemiSWEET. (**a**) Crystal structure of *L. biflexa* SemiSWEET (PDB 4QNC), showing the slight bending of TM2. The 3TM repeats are coloured as in Fig. 1c. The residues constituting the gates are shown as stick models. (**b**) Close-up view of the extracellular gate of *L. biflexa* SemiSWEET. The hydrogen-bonding and salt bridge interactions are depicted as black dotted lines. (**c**) Close-up view of the intracellular gate of *L. biflexa* SemiSWEET.

Notably, both the extracellular and intracellular gates are ‘closed’ in the previously reported occluded conformation of *L. biflexa* SemiSWEET (LbSemiSWEET) (Fig. 5a–c and Supplementary Fig. 3)^25^, where the amino acid residues constituting these two gates are completely conserved. A structural comparison of the present two distinct conformations of the EcSemiSWEET and the occluded conformation of the LbSemiSWEET revealed a slight bend at TM2 in the LbSemiSWEET (Fig. 5a–c). This bend allows the closing of both the extracellular and intracellular gates in the occluded conformation. This observation suggested that the helix bundle constituting each piece of the ‘binder clip’ does not move as an exact rigid body, but it may allow a slight bend in TM2. The extracellular and intracellular gates might associate with each other through this elasticity.

### Transport cycle of SemiSWEET

**Figure 6:**
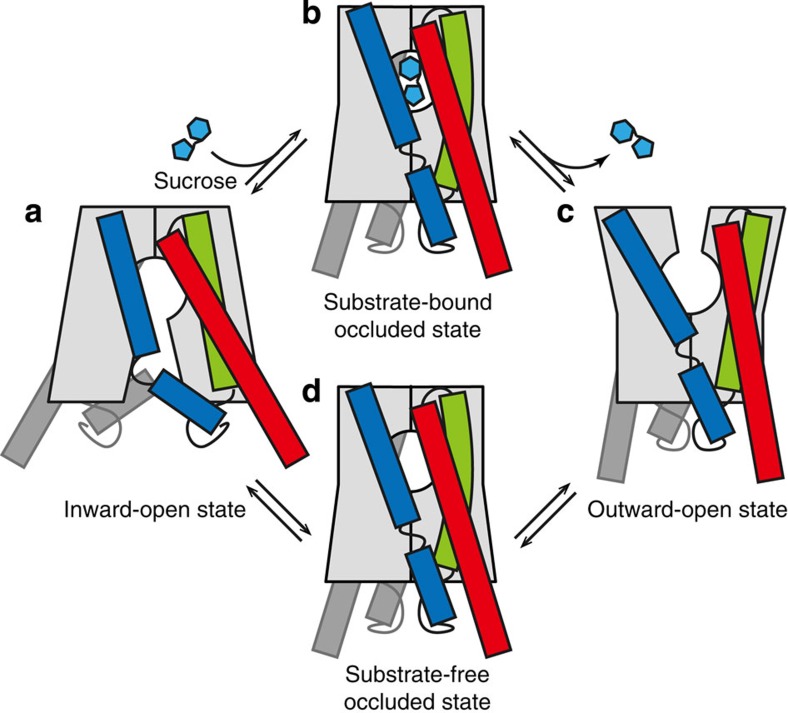
Transport cycle of SemiSWEET. (**a**–**d**) Schematics of the SemiSWEET dimer in the inward-open (**a**), substrate-bound occluded (**b**), outward-open (**c**) and substrate-free occluded (**d**) states, viewed parallel to the membrane. (**a**,**c**) are drawn based on the present crystal structures.

The present crystal structures and functional analysis allowed us to propose the transport cycle of EcSemiSWEET (Fig. 6a–d and Supplementary Movie 1). In the present structure in the inward-open conformation, the putative substrate-binding pocket is occupied by the monoolein molecule mimicking the substrate sugar, indicating that the observed structure represents an inward-open, substrate-binding state. The PQ-loop motif serves as a hinge that enables the binder clip-like motion of SemiSWEET to transit towards the outward-open state. Along with this transition, the slight bend of TM2 might close the intracellular gate, leading to the occluded state (Fig. 6b), while the closely associated opening/closing of the gates would prevent the formation of an open channel. Subsequently, the opening of the extracellular gate allows the substrate to exit (Fig. 6c). As the inward-open and outward-open conformations were simultaneously captured in Crystal-II in the absence of any sugars (Supplementary Fig. 2), the transition between these two conformations could spontaneously occur even without any substrates (Fig. 6d). These sequential conformational changes allow the small amphipathic molecules that can be accommodated in the central pocket to permeate through the membrane, while preventing the leakage of the larger molecules or ions.

In summary, the current structures and functional analyses revealed the molecular detail of ‘alternating access’ by the SWEET and PQ-loop transporters mediated by the binder clip-like motion. The mechanistic insight presented here will aid further experiments towards understanding the substrate selectivity, transport kinetics and regulatory mechanism of the plant SWEETs and the malfunction of the human PQ-loop transporters.

## Methods

### Protein expression and purification

The SemiSWEET gene (GI: 446301855) was amplified from *E. coli* K-12 genomic DNA (Strain: JCM 20135) and subcloned into a modified pET vector. The resulting plasmid, encoding SemiSWEET-LESSGENLYFQGQFTS-H_8_, was transformed into *E. coli* Rosetta 2 (DE3) cells and protein expression was induced with 0.2 mM isopropyl β-D-thiogalactopyranoside when the culture reached an OD_600_=0.6. After growth for 20 h at 20 °C, the cells were pelleted and resuspended in buffer containing 50 mM Tris-HCl (pH 8.0), 150 mM NaCl and 0.1 mM phenylmethylsulfonyl fluoride, and then disrupted using a Microfluidizer processor (Microfluidics) with three passes at 15,000 psi. Cell debris was removed by low-speed centrifugation at 10,000*g* for 10 min and the membrane fraction was collected by ultracentrifugation at 138,000*g* for 1 h. The membrane fraction was solubilized in buffer, containing 20 mM Tris-HCl (pH 8.0), 150 mM NaCl, 10 mM imidazole, 2% dodecyl-β-D-maltopyranoside (DDM) and 0.4% cholesteryl hemisuccinate (CHS), for 90 min at 4 °C. Insoluble components were removed by ultracentrifugation at 138,000*g* for 30 min and the supernatant was mixed with Ni-NTA resin (Qiagen) for 90 min. The resin was washed with 20 mM Tris-HCl (pH 8.0), 150 mM NaCl, 50 mM imidazole, 0.05% DDM and 0.01% CHS, and the protein was eluted with the same buffer supplemented with a final concentration of 300 mM imidazole. The eluate was treated with tobacco etch virus protease to cleave the His_8_-tag, dialysed overnight against the imidazole-free buffer and then reloaded onto the Ni-NTA resin to remove the cleaved tag and the protease. The flow-through fraction containing SemiSWEET was concentrated to ~5 mg ml^−1^ with a 30-kDa concentrator (Millipore) and was further purified by chromatography on a Superdex 200 Increase 10/300 gel filtration column (GE Healthcare), in buffer containing 10 mM Tris-HCl (pH 8.0), 100 mM NaCl, 0.05% DDM and 0.01% CHS. The purified protein was concentrated to 15 mg ml^−1^, flash frozen in liquid nitrogen and stored at −80 °C until crystallization. The SeMet-labelled SemiSWEET protein was expressed in *E. coli* B834 (DE3) cells and purified by the same procedure as for the native protein.

### Crystallization

For crystallization, the purified samples were reconstituted into the LCP of 1-oleoyl-R-glycerol (monoolein) at a protein to lipid ratio of 2:3 (w/w), using the two-syringe mixing method^30^. For the sandwich-drop crystallization, aliquots of the protein-LCP mixture were dispensed onto 96-well glass plates and overlaid with the precipitant solution, using a mosquito LCP (TTP LabTech). For hanging-drop crystallization, the protein-LCP drops were manually spotted onto siliconized glass coverslips and overlaid with the precipitant solutions, and then the coverslips were placed upside down onto 24-well plates and sealed with each well containing 300 μl of reservoir solution, with the same composition as that of the precipitant solution. The native protein was crystallized under two similar conditions (Crystal-I and Crystal-II). Crystal-I was grown in sandwich-drop plates, with 50 nl protein-LCP drops overlaid with 700 nl precipitant solution, which consisted of 28% PEG550MME, 100 mM Tris-HCl (pH 8.0), 350 mM MgSO_4_ and 3% galactose. Crystal-II was grown in hanging-drop plates, with 50 nl protein-LCP drops overlaid with 800 nl precipitant solution, which consisted of 23% PEG550MME, 100 mM Tris-HCl (pH 8.0), 350 mM NH_4_-citrate and 3% dimethyl sulfoxide. The SeMet-labelled protein was crystallized under conditions similar to those for Crystal-I, using a precipitant solution consisting of 23%–30% PEG550MME, 100 mM Tris-HCl (pH 8.0) and 150–200 mM (NH_4_)_2_SO_4_. All of the crystals were harvested and flash cooled in liquid nitrogen for data collection.

### Data collection and structure determination

X-ray diffraction data were collected at the SPring-8 beamline BL32XU, using the helical data collection method with a 1 × 10 μm (width × height) microbeam. Diffraction data were processed using XDS^31^. For Crystal-I, the structure was determined by the single-wavelength anomalous dispersion method, using the merged data from six SeMet-labelled crystals. Se sites were determined using SHELXD^32^ and initial phases were calculated using AutoSHARP^33^. The model was automatically built using PHENIX AutoBuild^34^,^35^. The obtained model was transferred to the native Crystal-I data and then iteratively rebuilt and refined using COOT^36^ and PHENIX, respectively. For Crystal-II, the structure was determined by molecular replacement in PHASER^37^, using the EcSemiSWEET monomer of Crystal-I as a search model. The resulting model was manually rebuilt using COOT and refined using REFMAC^38^ or PHENIX (Table 1 and Supplementary Fig. 7).

### Liposome [^14^C]-sucrose uptake assay

The purified SemiSWEET protein was reconstituted into liposomes by the following procedure. *E. coli* Polar Lipid Extract (Avanti) was dissolved in chloroform and dried into a thin film. This film was then resuspended to a final concentration of 20 mg ml^−1^ in buffer containing 10 mM Tris-HCl (pH 8.0) and 100 mM NaCl, and sonicated for 1 min to obtain the liposome solution. The purified protein was added to the liposome solution at a lipid to protein ratio of 100:1 (w/w), while 0.05% DDM was also added to avoid protein denaturation. The protein–liposome mixture was freeze–thawed three times for full reconstitution and then sonicated for 1 min for unilamellar vesicle formation. Protein-free liposomes were prepared by a similar procedure, except that the protein solution was replaced with the buffer used for the final purification step.

The time-dependent [^14^C]-sucrose uptake assay was initiated by mixing the liposome solution with an equal volume of the extraliposomal solution, consisting of 10 mM Tris-HCl (pH 8.0), 100 mM NaCl and 10 mM [^14^C]-sucrose (1 μCi ml^−1^). After the reaction at 37 °C, the liposomes were isolated by gel-filtration with Sephadex G-50 (GE Healthcare) and the radioactivity of the incorporated [^14^C]-sucrose was measured by liquid scintillation counting. The concentration-dependent [^14^C]-sucrose uptake was measured by a similar procedure, with different concentrations of the extraliposomal [^14^C]-sucrose. For mutational analyses, mutations were introduced by a PCR-based method. The mutant proteins were expressed, purified and reconstituted into liposomes, and the transport activities were measured by a similar procedure as for the wild type.

## Author contributions

Y.L. expressed and purified EcSemiSWEET for crystallization, collected the diffraction data, solved the structures and performed the functional analyses in liposomes. K.Y. assisted and performed data processing. Y.L., T.N., R.I. and O.N. wrote the manuscript. O.N. directed and supervised all of the research.

## Additional information

**How to cite this article**: Lee, Y. *et al*. Structural basis for the facilitative diffusion mechanism by SemiSWEET transporter. *Nat. Commun.* 6:6112 doi: 10.1038/ncomms7112 (2015).

**Accession codes:** The atomic coordinates and structure factors of EcSemiSWEET have been deposited in the Protein Data Bank under the accession codes 4X5M (Crystal-I) and 4X5N (Crystal-II).

## Supplementary Material

Supplementary Figures Supplementary Figures 1-7

Supplementary Movie 1 State transition induced by the PQ-loop motif

## Figures and Tables

**Table 1:** Data collection, phasing and refinement statistics.

	**Native Crystal-I**	**SeMet Crystal-I**	**Native Crystal-II**
*Data collection*			
Space group	*P*2_1_2_1_2	*P*2_1_2_1_2	*C*2
Cell dimensions			
*a*, *b*, *c* (Å)	53.7, 102.1, 59.0	53.8, 101.0, 58.65	118.0, 34.6, 123.2
*α*, *β*, *γ* (°)	90.0, 90.0, 90.0	90.0, 90.0, 90.0	90.0, 102.9, 90.0
Wavelength (Å)	1.0000	0.9792	1.0000
Resolution (Å)	50.0–2.00 (2.07–2.00)	50.0–2.60 (2.69–2.60)	50.0–3.00 (3.11–3.00)
*R*_merge_	0.139 (1.809)	0.333 (3.812)	0.112 (0.738)
*I*/σ*I*	17.7 (2.0)	15.1 (1.4)	8.2 (1.7)
Completeness (%)	99.9 (100.0)	99.9 (100.0)	98.0 (98.4)
Redundancy	19.53 (18.01)	46.8 (31.3)	3.3 (3.2)
			
*Refinement*			
Resolution (Å)	50–2.00		50–3.00
No. reflections	22,575		9,943
*R*_work_/*R*_free_	19.7/22.4		28.1/32.8
No. atoms			
Protein	2,077		2,679
Lipid/ion	259		
Water	66		
*B*-factors			
Protein	36.02		67.17
Lipid/ion	55.07		
Water	47.37		
R.m.s deviations			
Bond lengths (Å)	0.0024		0.0035
Bond angles (°)	0.639		0.713
Ramachandran plot			
Favored (%)	100.0		97.8
Allowed (%)	0.0		2.2
Outliers (%)	0.0		0.0

SeMet data were collected from six crystals and others were from one crystal.

The values in parentheses are for the highest-resolution shell.
