# Supplementary material for: Structural basis for the facilitative diffusion mechanism by SemiSWEET transporter
Source: Nat Commun. 2015 Jan 19;6:6112. doi: 10.1038/ncomms7112 (PMC4309421; doi:10.1038/ncomms7112)
Supplement: Supplementary Figures — 1-7 [file ncomms7112-s1.pdf]

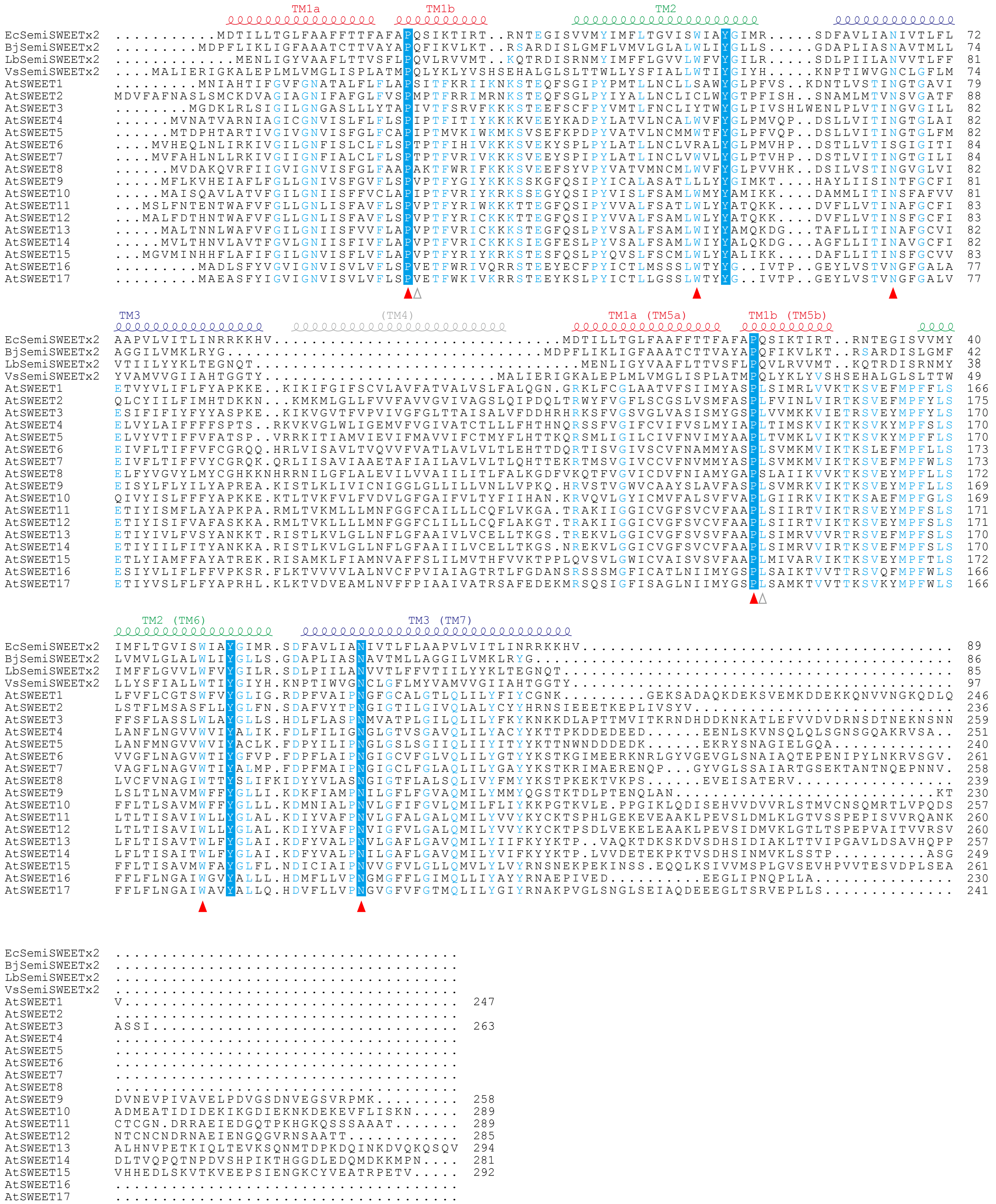

Supplementary Figure 1 | Multiple alignment of SemiSWEET and SWEET proteins. Sequence alignment of EcSemiSWEET, BjSemiSWEET, *Leptospira biblexa* (Lb) SemiSWEET, *Vibrio sp.* (Vs) SemiSWEET, and *Arabidopsis* (At) SWEET1–17, created using ClustalW2 and manually adjusted. The sequences of the SemiSWEETs are duplicated to align with the two 3TM repeats in the SWEETs, and are thus designated as "(species name) SemiSWEETx2". The secondary structure of EcSemiSWEET is shown above the sequences. Predicted TM helices (TM4 to TM7) in the SWEETs are shown in parentheses. The positions of the conserved functional residues are indicated by red arrows. The positions of the glutamine residues in the PQ motif are indicated by white arrows. The glutamine residues are not conserved in the SWEETs.

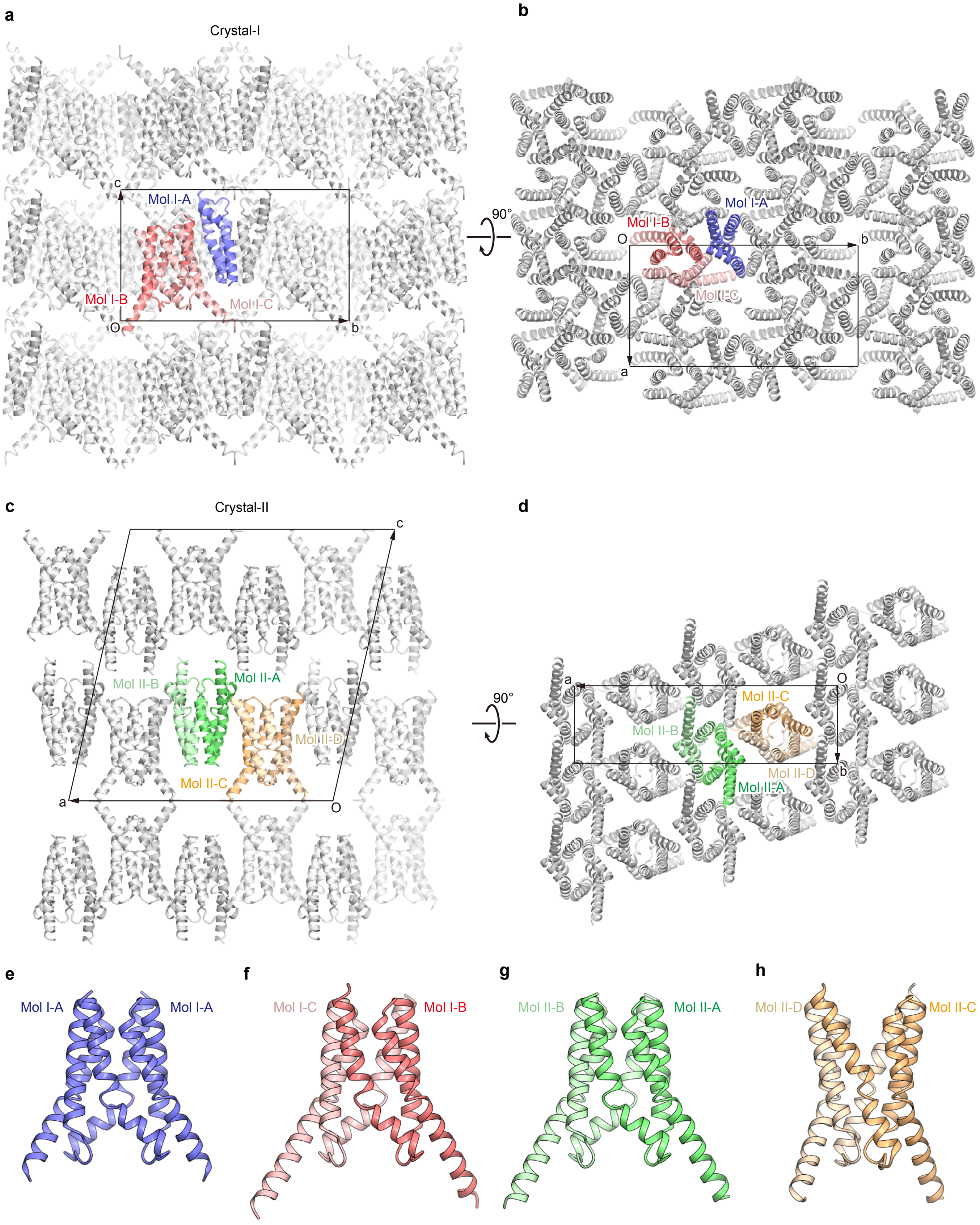

Supplementary Figure 2 | Dimerization of SemiSWEET. (**a**, **b**) The crystal packing of Crystal-I, viewed parallel (**a**) or perpendicular (**b**) to the membrane layer. Chains A, B and C are labeled as Mol I-A, I-B and I-C, and colored blue, red and pink, respectively. (**c**, **d**) The crystal packing of Crystal-II, viewed parallel (**c**) or perpendicular (**d**) to the membrane layer. Chains A, B, C and D are designated as Mol II-A, II-B, II-C and II-D, and colored green, light green, orange and light orange, respectively. For each panel, the two crystallographic axes parallel to the plane of the view are depicted as arrows. (**e**–**h**) Dimeric assemblies of SemiSWEET. The dimers I-A/I-A (**e**), I-B/I-C (**f**) and II-A/II-B (**g**) adopt almost identical conformations (the inward-open state), whereas the dimer II-C/II-D (**h**) adopts a distinct conformation (the outward-open state).

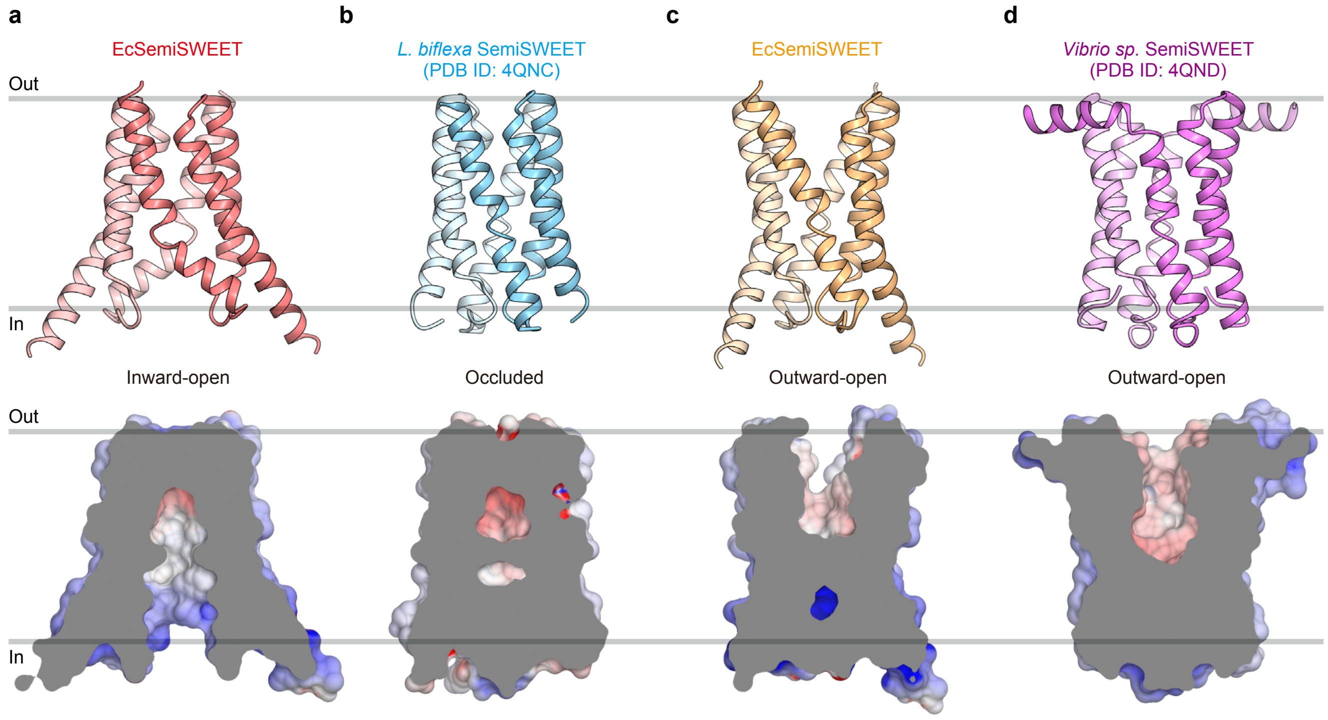

Supplementary Figure 3 | Different conformations of SemiSWEETs. (**a**–**d**) Crystal structures of the inward-open EcSemiSWEET (**a**), the occluded *L. biflexa* SemiSWEET (PDB 4QNC) (**b**), the outward-open EcSemiSWEET (**c**) and the outward-open *Vibrio sp.* SemiSWEET (PDB 4QND) (**d**). Cut-away surface is shown on the bottom of each structure.

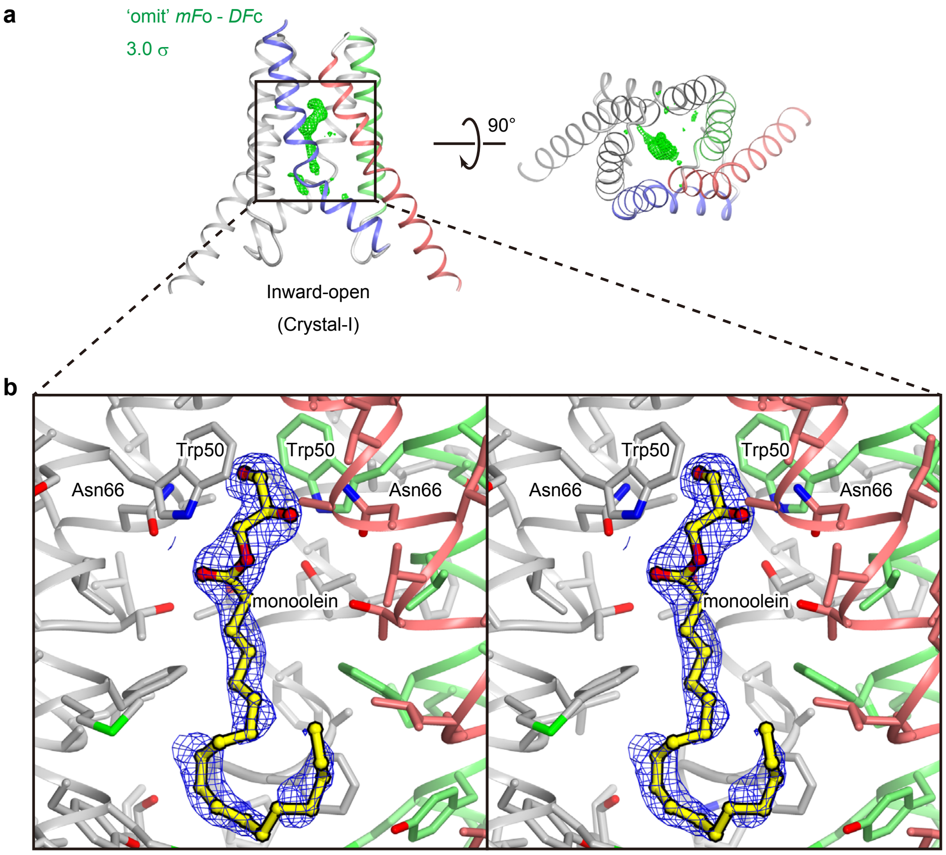

Supplementary Figure 4 | Monoolein molecule at the putative substrate-binding pocket. (**a**) The 'omit' $mF_O - DF_C$ electron density map of the inward-open dimer from Crystal-I, calculated without the monoolein molecule in the pocket, contoured at 3.0 σ. Strong electron densities were observed in the putative-substrate binding pocket. (**b**) Stereo view of the modeled monoolein molecule. The $2mF_O - DF_C$ electron density map is contoured at 1.0 σ. The TM1 helix in front of the monoolein is omitted for clarity.

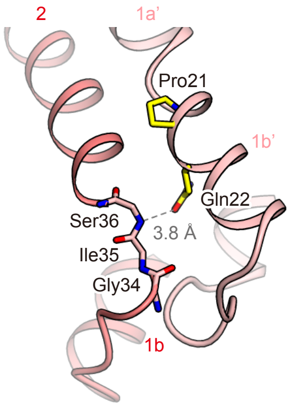

Supplementary Figure 5 | PQ-loop motif of the outward-open conformation. In the outward-open conformation, the rotamers of the Gln22 sidechains are slightly different between the two protomers, probably due to the low data quality of Crystal-II. While the Gln22 side chain of one protomer hydrogen bonds with the adjacent Ser36 main chain as shown in Fig. 3e, the equivalent Gln22 residue of the other protomer is not within the hydrogen-bonding distance with the Ser36 main chain.

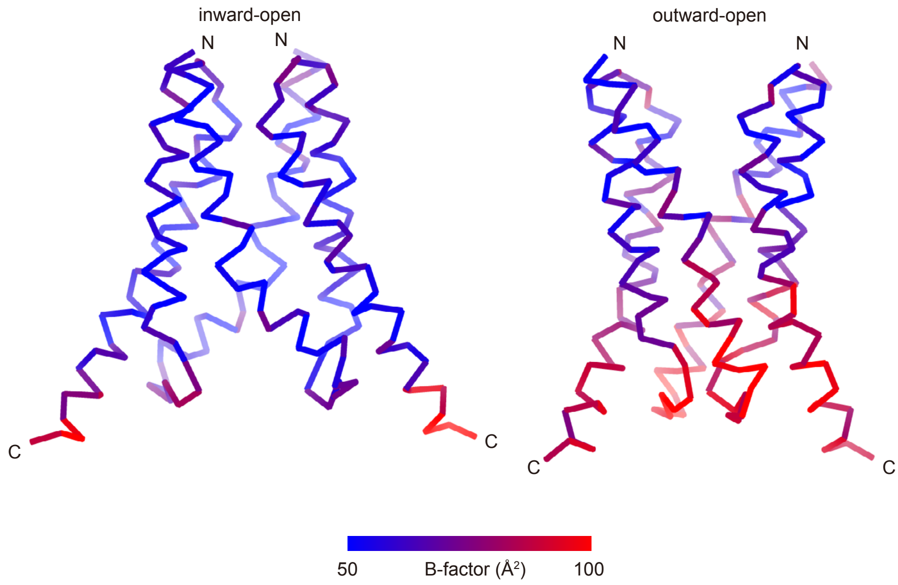

Supplementary Figure 6 | Local B-factors of Crystal-II. Main chain traces of the two SemiSWEET dimers from Crystal-II, colored according to the local B-factor values. The coloring scheme is shown at the bottom.

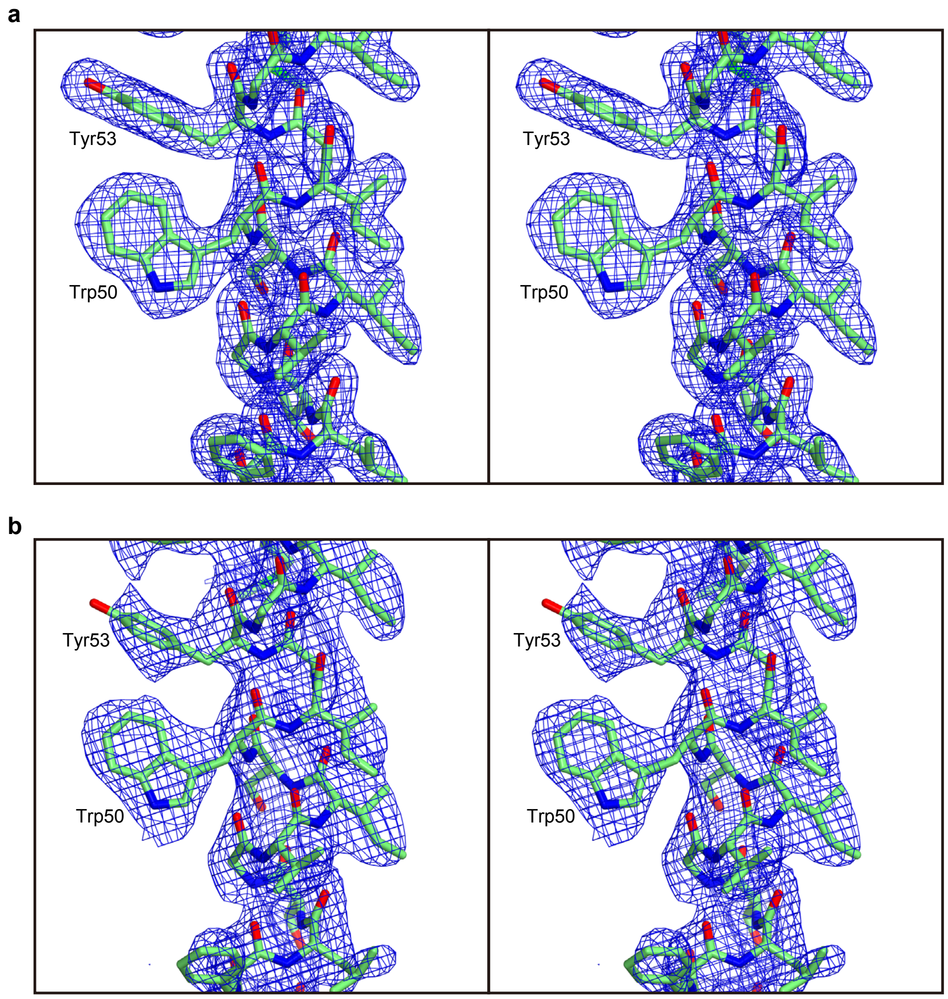

Supplementary Figure 7 | Examples of electron density. (**a**, **b**) Stereo views of the $2mF_O - DF_C$ electron density maps of the TM2 helix from Crystal-I (**a**) and Crystal-II (**b**), contoured at 1 σ.
